# Integrative analysis of transcriptomic data for identification of T-cell activation-related mRNA signatures indicative of preterm birth

**DOI:** 10.1038/s41598-021-81834-z

**Published:** 2021-01-27

**Authors:** Jae Young Yoo, Do Young Hyeon, Yourae Shin, Soo Min Kim, Young-Ah You, Daye Kim, Daehee Hwang, Young Ju Kim

**Affiliations:** 1 Department of Obstetrics and Gynecology, College of Medicine and Ewha Medical Institute, Ewha Womans University, Seoul, 07804 Republic of Korea; 2 School of Biological Sciences, Seoul National University, Seoul, 08826 Republic of Korea; 3 Ewha Medical Institute, Ewha Medical Center, Ewha Womans University, Seoul, 07804 Korea; 4 Internship in Ewha Womans University Mokdong Hospital, College of Medicine, Ewha Womans University, Seoul, 07804 Republic of Korea

**Keywords:** Molecular biology, Medical research, Risk factors

## Abstract

Preterm birth (PTB), defined as birth at less than 37 weeks of gestation, is a major determinant of neonatal mortality and morbidity. Early diagnosis of PTB risk followed by protective interventions are essential to reduce adverse neonatal outcomes. However, due to the redundant nature of the clinical conditions with other diseases, PTB-associated clinical parameters are poor predictors of PTB. To identify molecular signatures predictive of PTB with high accuracy, we performed mRNA sequencing analysis of PTB patients and full-term birth (FTB) controls in Korean population and identified differentially expressed genes (DEGs) as well as cellular pathways represented by the DEGs between PTB and FTB. By integrating the gene expression profiles of different ethnic groups from previous studies, we identified the core T-cell activation pathway associated with PTB, which was shared among all previous datasets, and selected three representative DEGs (*CYLD*, *TFRC*, and *RIPK2*) from the core pathway as mRNA signatures predictive of PTB. We confirmed the dysregulation of the candidate predictors and the core T-cell activation pathway in an independent cohort. Our results suggest that *CYLD*, *TFRC*, and *RIPK2* are potentially reliable predictors for PTB.

## Introduction

Preterm birth (PTB) is the birth of a baby at less than 37 weeks of gestation, as opposed to the usual about 40 weeks, called full term birth (FTB)^1^. The rate of PTB is 10.6% of births worldwide^2,3^. In Korea, the rate of PTB has steadily increased from 4.8% in 2006 to 7.2% in 2016^4^. Preterm babies show adverse short- and long-term health outcomes. Short-term outcomes include neonatal death, neurodevelopmental disabilities, and under five-year mortality^5^. Long-term problems include increased risk of hypertension, type 2 diabetes, cardiovascular disease, chronic kidney disease, asthma, and neurocognitive disorders^6–8^. Owing to these adverse outcomes, it is important to diagnose PTB as early as possible, followed by protective interventions. However, the clinical criteria for PTB diagnosis remain inaccurate until the onset of labor.

The clinical criteria for the diagnosis of PTB have been developed based on various non-modifiable and modifiable factors, and clinical conditions that contribute to the risk of PTB. Non-modifiable factors include PTB history, maternal age, multiple pregnancies, obstetric complications, and genetic factors (genetic alterations and aberrant DNA methylation)^9–11^. The modifiable factors include nutrition, maternal reproductive tract infections, behavioral factors, and emotional status^3^. Further, clinical conditions leading to PTB include decidual hemorrhage, uterine distension, maternal distress that leads to increased prostaglandin production, uterine infection, inflammation, and premature rupture of membrane^9^. Since little is known about the etiology of PTB, the effectors of PTB remain elusive, precluding the use of these conditions as predictors of PTB risk.

Therefore, significant efforts have been made to systematically identify molecular signatures that can be used to diagnose PTB with high accuracy, together with the clinical criteria mentioned above. The Preterm Birth International Collaborative (PREBIC) reviewed and identified 116 predictors for PTB from blood, amniotic fluid, and cervicovaginal fluid from 217 studies published between 1965 and 2008^12^. However, PREBIC did not find a single molecule that reliably predicts PTB. Several studies performed transcriptomics or proteomics using whole blood, placenta, amniotic fluid, or cervicovaginal fluid from PTB patients with different sampling time, cohort size, ethnic group, and technical platforms. Heng et al.^13^ compared the gene expression profiles of whole blood samples from 75 Australian PTB patients and 79 full-term birth (FTB) controls. Paquette et al.^14^ also performed mRNA sequencing analysis of whole blood samples from 15 PTB patients and 23 FTB controls (mixed ethnic groups). However, there is the lack of shared molecular signatures due to the heterogeneity originating from the above differences.

Here, we initially performed mRNA sequencing analysis in five Korean PTB patients and five FTB controls and identified the differentially expressed genes (DEGs) between them. These DEGs, and the cellular pathways enriched by them may serve as candidate predictors for PTB in the Korean population. By integrating two previously reported gene expression profiles from different ethnic groups, we identified a PTB-associated cellular pathway shared among the different datasets and selected representative DEGs of the shared cellular pathway as reliable PTB predictor candidates. We finally confirmed the dysregulation of the selected candidates and the shared pathway in an independent cohort of 83 Korean PTB patients and 113 FTB controls, to support the validity of the PTB predictor candidates.

## Results

### DEGs between PTB and FTB groups

We collected whole blood samples from the subjects at clinical sign with delivery in the discovery cohort (5 PTB patients and 5 FTB controls) and the validation cohort (83 PTB patients and 113 FTB controls). Singleton pregnant women diagnosed with preterm labor (PTL) and/or premature preterm rupture of membranes (pPROM) were included as PTB patients in the cohorts. Women with multiple births, major birth defects, and pregnancy complications such as preeclampsia, gestational diabetes were excluded. Clinical parameters for the subjects in the discovery and validation cohorts were summarized in Table S1. In these cohorts, age, parity, and gravidity showed no significant differences between PTB patients and FTB controls while gestational age, birth weight, and APGAR scores (1 and 5 min) are significantly different. Moreover, PTB patients showed higher rates of chorioamnionitis than FTB controls. To identify molecular signatures associated with PTB, transcriptomic or proteomic analysis has been applied to whole blood samples (Australian cohort^13^ and a cohort of mixed ethnic groups^14^) or placenta-related tissue samples (placenta^15^ and tissue biopsies^16^). In this study, we used whole blood samples, rather than placenta-related tissues, to focus on non-invasive molecular predictors of PTB, which can be more efficient for fast screening purposes in regular check-ups during pregnancy than invasive predictors from placenta-related tissues^17^.

**Figure 1:**
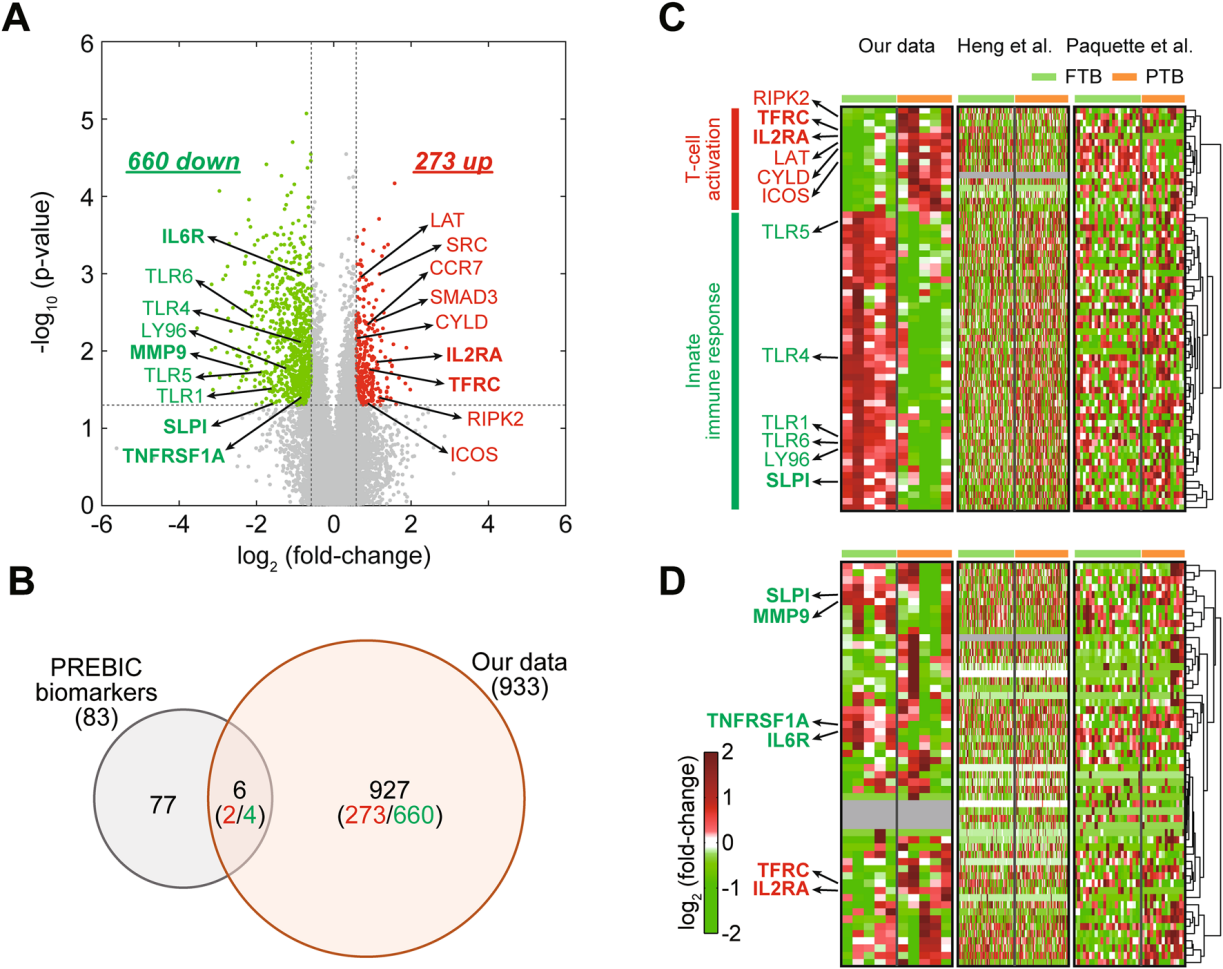
Differentially expressed genes (DEGs) between PTB (preterm birth) and FTB (full-term birth) groups. (**A**) Volcano plot for the comparison of PTB with FTB. Dotted lines represent the cutoffs for log_2_-fold-change and adjusted *p* value. Red and green dots denote the 273 upregulated and 660 downregulated genes, respectively. The representative up- and downregulated genes involved in T-cell activation (red) and innate immune response (green), respectively, are indicated by arrows. The six genes that overlapped with the 83 PREBIC PTB predictors are highlighted in bold. (**B**) Venn diagram showing the relationship between the DEGs and the PREBIC PTB predictors. Numbers of upregulated (red) and downregulated (green) genes in the sections of the Venn diagram are shown. (**C**,**D**) Heat maps showing expression changes of up or downregulated genes involved in T-cell activation and innate immune response, respectively and the 56 genes expressed (**C**) in at least one dataset (**D**) in all the three datasets. The color bar represents the gradient of log_2_-fold-change of mRNA expression levels in the samples with respect to the median mRNA expression levels.

We performed mRNA sequencing analysis of the whole blood samples collected from the discovery cohort. The numbers of measured and mapped reads for mRNA sequencing data are summarized in Table S2. Principal component analysis showed that the PTB patients could be distinguished from the FTB controls using the mRNA sequencing data (Fig. S1). To identify the genes that can distinguish the PTB patients and FTB controls, we first identified 15,662 genes expressed in at least half (n ≥ 3) of either PTB or FTB group. Among these expressed genes, we then identified 933 DEGs (273 upregulated and 660 downregulated genes) between PTB and FTB groups using an integrative statistical testing method previously reported (Fig. 1A; Table S3)^18^. The PREBIC summarized 116 PTB predictors reported in the 217 previous studies^12^, including 83 genes/proteins, their variants (truncated or post-translationally modified proteins), and steroid/metabolite hormones (e.g., cortisol, dopamine, dehydroepiandrosterone, or estrogen/progesterone). Among our 933 DEGs, only six genes (upregulated: *TFRC* and *IL2RA*; and downregulated: *TNFRSF1A*, *MMP9*, *SLPI*, and *IL6R*) overlapped with the 83 predictor genes previously identified by PREBIC (Fig. 1B). The PREBIC also listed 16 predictors that were most frequently reported (≥ 10 times) in the 217 studies^12^. Among the six overlapping genes, matrix metalloproteinase 9 (*MMP9*) belonged to the 16 most common predictors.

The small overlap between our DEGs and the previous predictors prompted us to investigate the consistency in dysregulations of these genes in the two other gene expression profiles of whole blood samples including an Australian cohort (75 PTB and 79 FTB)^13^ and a cohort of mixed ethnic groups (15 PTB and 23 FTB)^14^. According to the PREBIC, the 83 previous predictor genes were most significantly associated with immune responses^12^. Consistent with this finding, our DEGs included 46 and 16 genes involved in innate immune response and T-cell activation, respectively. These immune-related DEGs in the Korean PTB cohort showed no significant mRNA expression changes in the two other gene expression datasets (Fig. 1C). Among the 83 previous predictor genes, 56 genes expressed in at least one dataset also showed no strong consistency in their dysregulations across the different cohorts (Fig. 1D). These data suggest that heterogeneity exists in PTB-associated gene expression changes across the different cohorts, consistent with the previous finding from the integrative analysis performed by the PREBIC^12^.

### Cellular processes represented by the DEGs

**Figure 2:**
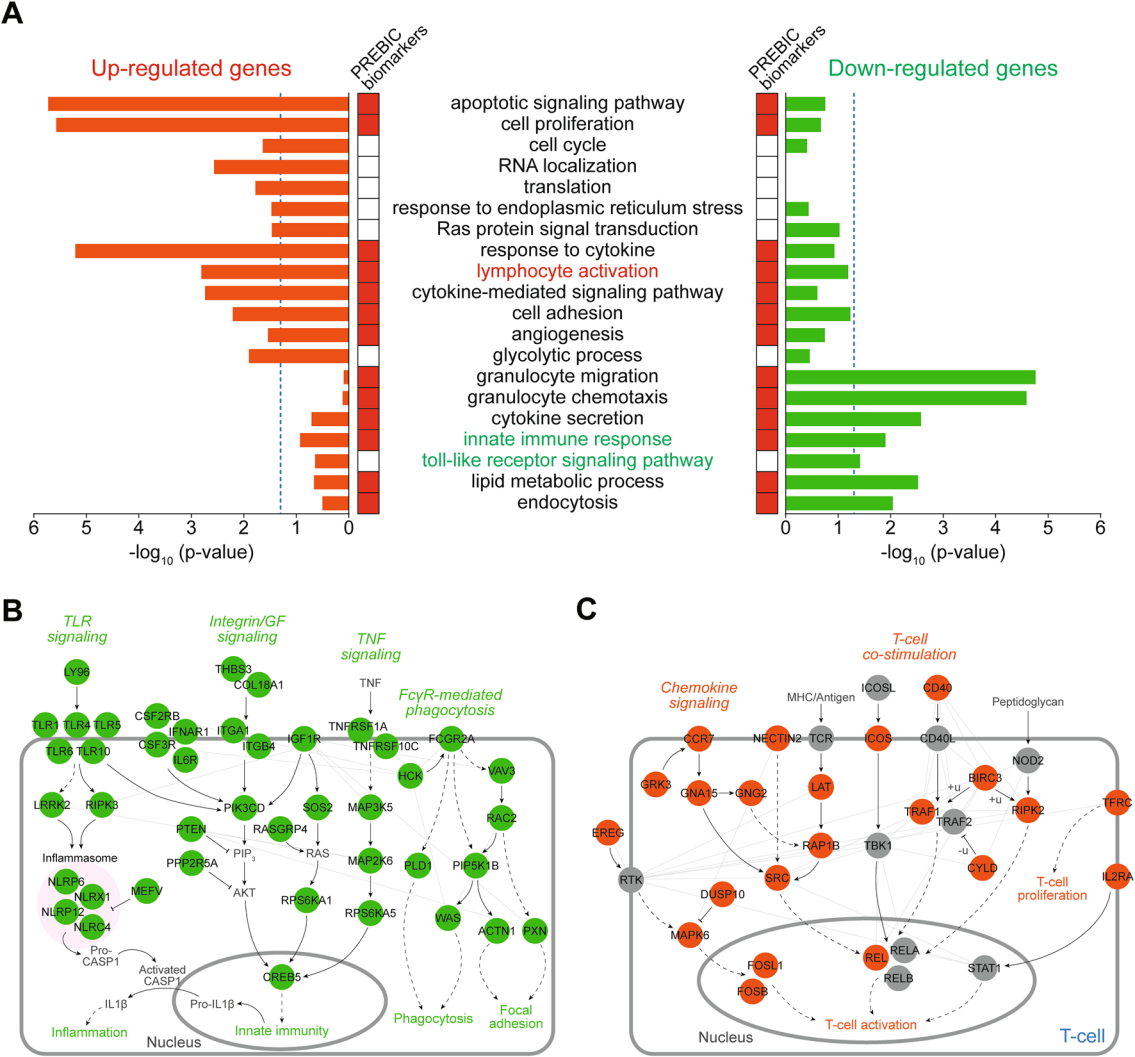
Cellular processes represented by the DEGs. (**A**) Gene ontology biological processes (GOBPs) enriched by the genes that were up- and downregulated in PTB compared to that in FTB. Significance (*p* value) of the GOBPs enriched by the upregulated (left bars) and downregulated (right bars) genes is displayed as − log_10_ (*p* value). GOBPs associated with the PREBIC PTB predictors are indicated in the red heat map (‘PREBIC PTB predictors’). (**B**,**C**) Network models describing interactions among the downregulated (**B**) or upregulated (**C**) genes involved in the processes related to innate immunity and T-cell activation, respectively. Node color represents upregulation (orange) or downregulation (green) in the comparison of PTB versus FTB. Gray nodes indicate the non-DEGs added in the network to increase connections among the DEGs. Solid and dashed edges represent direct and indirect activation (arrow) or repression (suppression symbol), respectively. Gray lines indicate protein–protein interactions. Thick gray lines denote the plasma and nuclear membranes.

We next examined cellular processes represented by the 933 DEGs in Korea PTB cohort by performing the enrichment analysis of gene ontology biological processes (GOBPs) for the 273 upregulated and 660 downregulated genes using the DAVID software^19^. The upregulated genes were mainly associated with the processes related to cell proliferation (cell cycle, cell proliferation, apoptotic signaling, response to ER stress, RAS signaling, and translation/RNA localization), adaptive immune response (lymphocyte activation, response to cytokine, and cytokine signaling), cell adhesion, angiogenesis, and carbohydrate metabolism (glycolytic process) (Fig. 2A, left). On the other hand, the downregulated genes were associated with the processes related to endocytosis, innate immune response (TLR signaling, granulocyte chemotaxis/migration, cytokine secretion), and lipid metabolism (lipid metabolic process; Fig. 2A, right). The 83 previous PTB predictor genes reported by the PREBIC were associated with a majority of these cellular processes (Fig. 2A, red heat map), indicating a strong overlap at the level of cellular pathways, unlike the small overlap at the gene level (Fig. 1B,C).

Interestingly, the adaptive immune response, and T-cell activation in particular, was upregulated in the Korean PTB patients, whereas the innate immune response was downregulated. To sort out this discrepancy at the molecular level, we reconstructed a network model describing interactions among the DEGs involved in the processes related to the immune responses. The network model showed that 1) TLR (*LY96*, *TLR*1/4/5/6/10, *LRRK2*, and *RIPK*) signaling, 2) TNF (*TNFRSF1A/10C*, *MAP3K5*, *MAP2K6*, and *RPS6KA*1/5) signaling, 3) Fc gamma receptor mediated phagocytosis (*FCGR2A*, *PLD1*, *VAV3*, *RAC2*, and *WAX*) and 4) integrin (*ITGA*1/4) and growth factor (*IGF1R*)-mediated cytoskeletal reorganization pathways were downregulated in PTB (Fig. 2B). In contrast, 1) T-cell activation (*LAT*, *NECTIN2*, *RAB1B*, *TFRC*, *CYL* and *RIPK2*) and co-stimulation (*CD40*, *ICOS*, and *TRAF1*) pathway, and 2) chemokine signaling in T-cells (*CCR7*, *GNA*2/15, and *GRK3*) were upregulated in PTB (Fig. 2C, red nodes). These data suggest that the number or activation of T-cells is increased in PTB, compared to that in FTB while the number or activation of innate immune cells is decreased.

### Core PTB-associated pathways identified through integration of gene expression profiles

**Figure 3:**
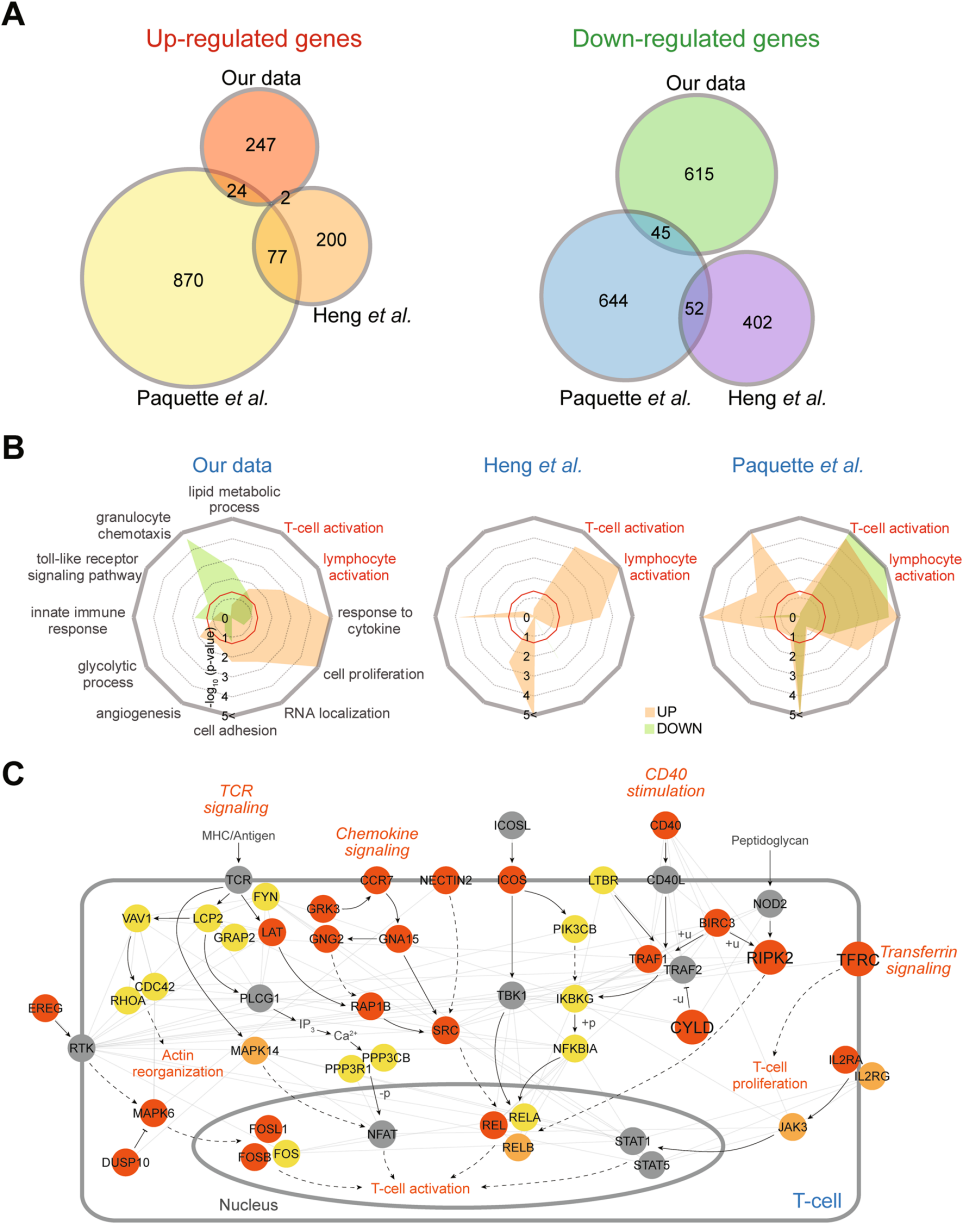
Core PTB-associated pathways identified by integration of the previous datasets. (**A**) Venn diagrams showing the relationships among upregulated (left) and downregulated genes (right) in PTB, compared to that in FTB, identified from our data and two previous datasets (Heng et al.^13^ and Paquette et al.^14^). (**B**) Radar charts showing the GOBPs enriched by our DEGs (left) in comparison with those enriched by the DEGs from Heng et al. (middle) and Paquette et al*.* datasets (right). Significance (*p* value) of the GOBPs enriched by the up- or downregulated genes is displayed as − log_10_(*p* value). (**C**) Network model describing interactions among the upregulated genes involved in the T-cell activation-associated processes in any of the three datasets. Dark orange, orange, and yellow nodes represent the upregulated genes identified from our data and Heng et al*.* and Paquette et al*.* datasets, respectively. See the legend of Fig. 2B,C for nodes, edges, and membranes.

The PREBIC performed an integrative analysis of PTB predictors reported in 217 studies to identify reliable predictors. Six gene expression profiles of the samples from PTB patients had been reported. To identify reliable PTB predictors among these profiles, we thus integrated our data with the aforementioned two gene expression profiles of whole blood samples from the Australian cohort^13^ and a cohort of mixed ethnic groups^14^. The other profiles were excluded due to possible misleading biases originating from non-blood samples (placenta^15^ and tissue biopsies^16^), or whole blood samples not collected before labor (4 days after birth^20^). Using the same method used for Korean PTB cohort, we identified 733 (279 up- and 454 downregulated) and 1712 (971 up- and 741 downregulated) DEGs from the Australian and mixed cohorts, respectively. Comparison of the DEGs revealed no significant overlaps between our data and the previous datasets (Fig. 3A), consistent with the findings from the PREBIC^12^ and the findings in Fig. 1B–D.

The lack of significant overlap at the gene level, and presence of a strong overlap at the pathway-level (Fig. 2A) prompted us to perform pathway-level integration of the three datasets, assuming that the non-overlapping DEGs can be involved in the same pathways. We performed the GOBP enrichment analysis for the up- and downregulated genes in the two previous datasets and then searched for the GOBPs shared in all three datasets. Among the GOBPs enriched by our DEGs, only T-cell (lymphocyte) activation was enriched consistently by the upregulated genes in all three datasets (Fig. 3B). Focusing on the T-cell activation, we next reconstructed an integrated network model describing interactions among the upregulated genes involved in T-cell activation-associated processes (T-cell/lymphocyte activation and response to cytokine) in all three datasets (Fig. 3C). The integrated network model showed that TCR, chemokine, CD40 stimulation, transferrin, and peptidoglycan signaling leading to T-cell activation were collectively supported by the upregulated genes in the three datasets. Since the TCR and chemokine signaling pathways are well known, we focused on the other signaling pathways. Among our upregulated genes, *CYLD*,* TFRC*, and *RIPK2* (Fig. 3C, large nodes) represented CD40 stimulation, transferrin, and peptidoglycan signaling pathways, respectively.

### PTB predictor candidates involved in the core PTB-associated pathway of T-cell activation

**Figure 4:**
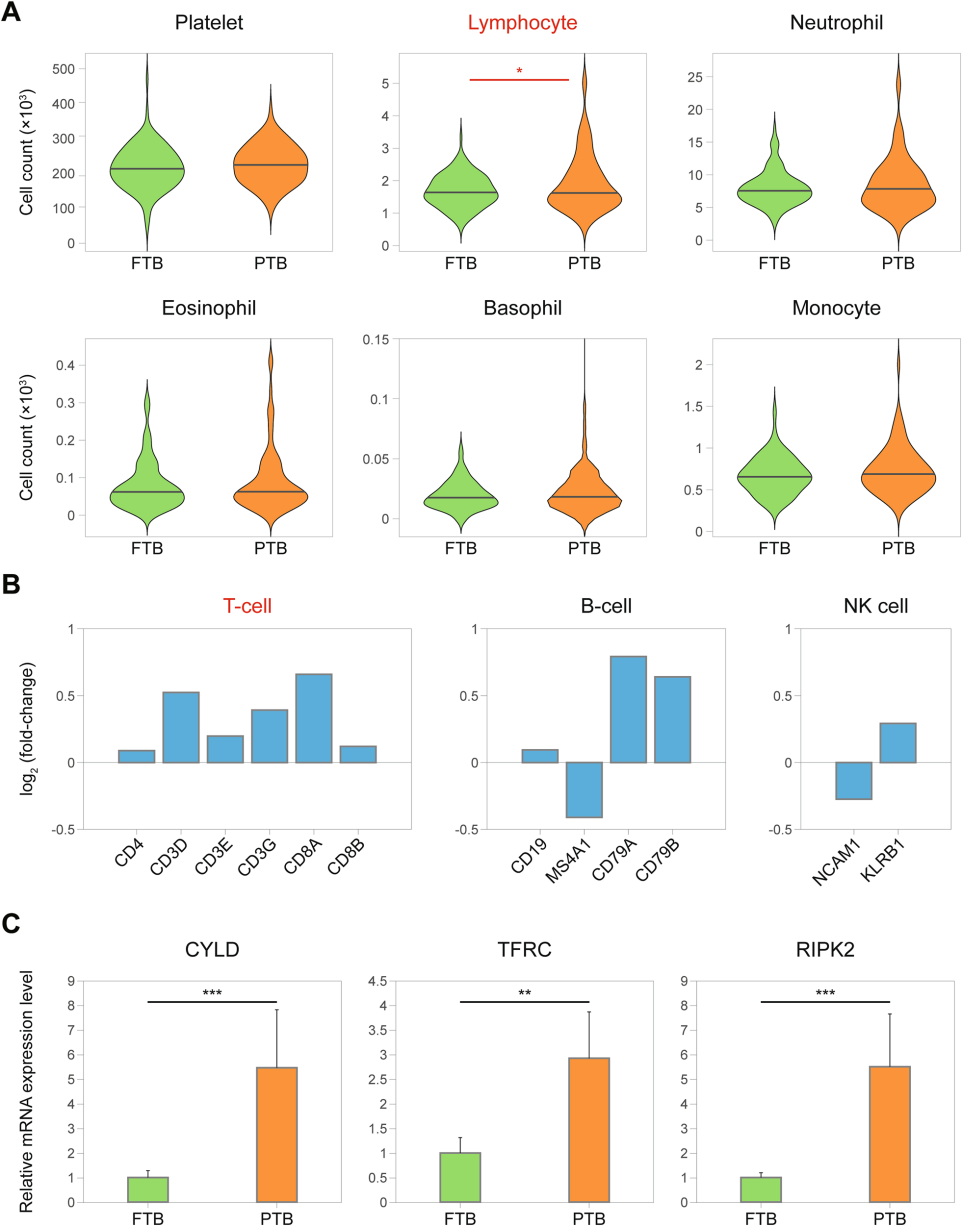
PTB predictor candidates involved in the core T-cell activation pathway. (**A**) The amount of the indicated immune cells in the whole blood samples from PTB patients and FTB controls in the validation cohort. In each violin plot, the middle line indicates 50th percentile of the indicated immune cell count. *p < 0.05 using unpaired t-test with Welch’s correction. (**B**) mRNA expression level of the indicated marker genes of T-cells, B-cells, and NK cells. Log_2_ (fold-change) of mRNA expression level between PTB and FTB (PTB/FTB) are displayed. (**C**) Relative mRNA expression level of *CYLD*, *TFRC*, and *RIPK2* in PTB samples (n = 30) with respect to those in FTB samples (n = 30). **p < 0.01, ***p < 0.001 by unpaired t-test with Welch’s correction; error bars, standard error of mean (SEM).

We next attempted to validate the core upregulated T-cell activation pathway in the validation cohort (113 FTB and 83 PTB). Overall, the mean maternal ages of FTB and PTB in the validation cohort were 33.4 and 32.9 years, respectively, and the mean gestational ages of FTB and PTB were 39 weeks and 31 weeks 6 days, respectively (Table S1B). We first examined the amount of six types of immune cells (lymphocyte, platelet, neutrophil, eosinophil, basophil, and monocyte) in the whole blood samples of PTB patients in the validation cohort using complete blood count (CBC) tests. Among the immune cells, only the count of lymphocytes in the blood was significantly (*p* < 0.05) increased in PTB, compared with that in FTB (Fig. 4A). Correspondingly, mRNA expression level of T-cell marker genes (CD4, CD8a/b, and CD3d/g/e) were increased in the blood samples of PTB (Fig. 4B). Finally, we checked for the upregulation of *CYLD*,* TFRC*, and *RIPK2* that represent the T-cell activation-associated signaling pathways, according to the integrated network analysis, using the whole blood samples from 30 PTB patients and 30 FTB controls in the validation cohort. Clinical characteristics of the subjects used for qRT-PCR analysis is summarized in Table 1. qRT-PCR analysis confirmed significant (*p* < 0.01) upregulation of the three representative genes in PTB, compared to that in FTB (Fig. 4C). Collectively, these data suggest that *CYLD*,* TFRC*, and *RIPK2* which represent core T-cell activation pathways can serve as reliable molecular candidates that can predict PTB.

**Table 1:** Clinical characteristics of subjects used for qRT-PCR analysis.

Items	Full term birth	Preterm birth	*p* value
	(n = 30)	(n = 30)	
Maternal age	33.5 ± 0.7	32.5 ± 0.7	0.315
**Education, n (%)**			0.145
Below high school	2 ( 6.7)	7 (23.3)	
College or more	28 (93.3)	23 (76.7)	
Pre-pregnancy BMI	22.3 ± 0.9	22.1 ± 1.2	0.900
**Parity, *n (%)***			0.498
Nulliparous	15 (50.0)	16 (53.3)	
Multiparous	15 (50.0)	14 (46.7)	
**Gravidity**			0.484
0	18 (60.0)	21 (70.0)	
≥ 1	12 (40.0)	9 (30.0)	
**Diagnosis**			
PTL		14 (46.7)	
PPROM		16 (53.3)	
FT	30 (100)		
Cervical length (cm)	2.5 ± 0.2	2.3 ± 0.2	0.480
Gestational age	39.0 ± 0.2	30.1 ± 0.6	< 0.001*
**Mode of delivery, n (%)**			0.209
Vaginal	20 (66.7)	15 (50.0)	
C-section	10 (33.3)	15 (50.0)	
**Chorioamnionitis, *n (%)***			< 0.001^†^
Positive	-	13 (43.3)	
Negative	30 (100)	17 (56.7)	
**Antibiotics, *n (%)***			0.060
Treatment	15 (50.0)	24 (80.0)	
No	15 (50.0)	6 (20.0)	
**Tocolytics**			< 0.001^†^
Treatment	-	14 (46.7)	
No	30 (100)	16 (53.3)	
Birth weight (g)	3241.0 ± 68.8	1758.4 ± 146.9	< 0.001*
**Gender, n (%)**			0.299
Male	14 (46.7)	19 (63.3)	
Female	16 (53.3)	11 (36.7)	
APGAR score 1 min	9.7 ± 0.2	6.6 ± 0.6	< 0.001*
APGAR score 5 min	9.9 ± 0.0	8.0 ± 0.5	< 0.001*

Data were presented as mean ± SE. *Student’s t-test, *p* < 0.05; ^†^χ^2^ test, *p* < 0.05. *BMI* body mass index, *PTL* Preterm labor, *pPROM* Preterm premature rupture of membrane, *FT* Full term.

## Discussion

According to the integrative analysis of mRNA and proteomic signatures performed by the PREBIC, the heterogeneity in the molecular signatures of PTB-associated changes is common, possibly due to molecular variations driven by ethnicity and/or heterogeneity in the initiation and progression of PTB. Correspondingly, our integrative analysis revealed that the mRNA signatures of PTB-associated expression changes were heterogeneous. To address this heterogeneity issue, we employed a pathway-level integration of PTB-associated molecular signatures to search for the core cellular pathways consistently enriched by the PREBIC PTB predictors and the DEGs in three different mRNA expression datasets. Despite the molecular-level heterogeneity across the datasets, this approach first revealed that innate and adaptive immune responses and lipid metabolism, which are suggested to be associated with the pathogenesis of PTB, were represented consistently by PREBIC predictors and our DEGs. Among the immune responses, this approach further identified the T-cell activation-associated pathways consistently upregulated in PTB in the three mRNA expression datasets. Finally, this approach identified three representative regulators (*CYLD*, *TFRC* and *RIPK2*) of these core T-cell activation pathways as the candidates of the whole blood molecular signatures indicative of PTB. Considering that these T-cell activation-associated pathways were consistently up-regulated in the three different cohorts, they may serve as the valid whole blood molecular signatures in other ethnic groups.

Although several studies have focused on establishing causal links between innate immune response and PTB, several studies have reported the association of T-cell activation, an adaptive immune response, with PTB. Arenas-Hernandez et al.^21^ found that activated T-cells were enriched at the maternal–fetal interface of PTB patients and progesterone treatment prevented PTB by attenuating T-cell related pro-inflammatory responses. Luciano et al.^22^ also showed the association of neonatal CD4 positive T-cell activation with PTB. Gomez-Lopez et al.^23^ showed enrichment of fetal T-cells and elevation of T-cell cytokines in amniotic fluid during preterm gestation, and that intra-amniotic administration of activated neonatal CD4+ T cells induced PTB in mice. These data suggest that T-cell activation is a core pathway associated with PTB pathogenesis.

We identified *CYLD*, *TFRC* and *RIPK2* as PTB predictor candidates representing the core T-cell activation pathway in PTB. *TFRC* mediates iron uptake in the placenta, and iron deficiency during pregnancy increases the risk of PTB^24^. *TFRC* is upregulated in the placenta of PTB patients^15^ and correspondingly hyper-methylated in the proximity of the *TFRC* gene at placental tissues from PTB patients^25^. Although *CYLD* and *RIPK2* play important roles in T-cell activation, unlike *TFRC*, no direct evidence has been reported for causal links of *CYLD* and *RIPK2* to PTB. An isoform of *RIPK2*, *RIPK1*, is upregulated in the whole blood samples of the patients with chorioamnionitis^26^. Thus, *CYLD* and *RIPK2* can be considered as novel regulatory factors whose functional roles in the pathogenesis of PTB should be further investigated using detailed mechanistic experiments.

In this study, whole blood samples were taken from women with signs of delivery (FTB) and with signs of preterm labor and/or premature preterm rupture of membrane (PTB). Accordingly, our molecular signatures reflect the status of the peripheral blood transcriptome after the symptoms are observed. The selected molecular signatures (*CYLD*, *TFRC* and *RIPK2*) can be considered as predictors of PTB, rather than biomarkers that can predict PTB before the onset of symptoms. Moreover, although the association of T-cell activation with PTB has been reported, as mentioned above, Gomez-Lopez et al.^27^ also demonstrated that the peripheral blood transcriptome signatures, including T-cell-associated mRNA signatures, changed during gestation in the maternal circulation throughout normal pregnancy. In both our discovery and validation cohorts, the gestational age for PTB patients was significantly smaller than that for FTB controls. Since the gestational age was not matched between PTB patients and FTB controls, the increase of T-cells and T-cell marker gene expression in the whole blood samples of PTB patients, compared to in those of FTB controls, could be affected by the difference in the gestational age. Accordingly, the up-regulation of our PTB predictor candidates (*CYLD*, *TFRC* and *RIPK2*) could be also affected by the gestational age difference.

We tested the validity of *CYLD*,* TFRC* and *RIPK2* as PTB predictors using a moderately sized Korean cohort (88 PTB patients and 118 FTB controls). The characteristics of these predictor candidates should be further examined in a larger cohort including the subtypes of PTB, such as early and very early PTBs. In this study, we measured the number of lymphocytes and mRNA expression levels of T-cell marker genes to evaluate the increased T-cell activation in PTB, compared to in FTB. However, the possibility that the activation status of T-cells can alter the peripheral blood transcriptome regardless of the number of T-cells cannot be excluded. Thus, whether these predictor candidates can contribute to the activation status of T-cells, as well as T-cell proliferation, should be investigated in detailed functional studies. Moreover, the validity of these predictor candidates should also be tested in multiple ethnic cohorts to examine whether they can serve as PTB predictors across diverse ethnic groups. Furthermore, the association of these predictor candidates with neonatal adverse outcomes of PTB should also be investigated to examine whether they have functional roles in the adverse effects of PTB. These studies can provide essential information regarding the utility of these predictor candidates in the diagnosis and therapy of PTB.

## Materials and methods

### Sample collection

We enrolled 88 PTB patients (who delivered preterm infants) and 118 FTB controls, who underwent prenatal examinations and delivery at the Ewha Womans University Medical Center between 2014 and 2019. These subjects were split into discovery (5 PTB and 5 FTB) and validation (83 PTB and 113 FTB) cohorts. Singleton pregnant women diagnosed with PTL and/or pPROM were included as PTB patients in the cohorts. PTL was diagnosed in patients with regular uterine contraction and 4 or more contractions in 20 min, or 8 or more in 60 min as detected by cardiotocography. Uterine activity was assessed by cardiotocography. To diagnose pPROM, we conducted sterile speculum exam for detecting amniotic fluid pooling in vaginal cavity, as well as nitrazine test for detecting rupture of the membranes. Gestational age was determined using the first day of the last menstrual period and ultrasound examination. The maternal characteristics (age, parity and body mass index) of the mother were analyzed at admission. When pregnant women were admitted to the hospital for delivery, blood samples were taken and stored at − 80 °C.

The present study was approved by the Institutional Review Board of the Ewha Womans University Hospital (EUMC 2018-07-007-010). The experiments were conducted in accordance with the approved guidelines, and informed consent was obtained from all the subjects.

### CBC tests

Whole blood samples taken from all subjects were immediately analyzed using CBC with XN-9000 (Sysmex, Kobe, Japan)/ADVIA 2120i (Siemens, Tarrytown, NY, USA) according to manufacturer’s protocol.

### Library preparation and data generation

#### RNA extraction

Total RNA was isolated from blood using TRIzol RNA Isolation Reagent (Life technologies, Carlsbad, CA, USA), and purified according to the manufacturer’s instructions. The RNA concentration was determined using a NanoDrop ND-1000 spectrometer, and the RNA integrity number (RIN) for each RNA sample was analyzed using a 2100 Bioanalyzer (Agilent Technologies, Santa Clara, CA, USA) and the Agilent RNA 6000 Nano Kit (Agilent Technologies, Santa Clara, CA, USA). The RINs of the ten samples were > 8, which is appropriate for RNA sequencing.

#### Stranded mRNA library construction

The isolated total RNA was processed for preparing RNA sequencing library using TruSeq stranded total RNA sample preparation kit (Illumina, San Diego, CA, USA) according to the manufacturer’s instructions. Briefly, rRNAs were depleted from 1 μg of total RNA using rRNA removal beads, followed by enzymatic shearing. After first and second strand cDNA synthesis, A-tailing and end repair were performed for ligation of proprietary primers that incorporate unique sequencing adaptors with index for tracking Illumina reads from multiplexed samples run on a single sequencing lane. For each library, an insert size of approximately 200 bp was confirmed by a bioanalyzer using an Agilent DNA Kit (Agilent Technologies, Santa Clara, CA) and quantification of library was measured by real-time PCR using CFX96 real-time system (BioRad, Hercules, CA, USA). Sequencing of each library was performed on an Illumina NextSeq500 and clusters of the cDNA libraries were generated on a TruSeq flow cell and sequenced for 76-bp paired end reads (2 × 76) with a TruSeq 200 cycle SBS kit (Illumina, San Diego, CA, USA). Raw data were processed, and base calling was performed using the standard Illumina pipeline [CASAVA ver. 1.8.2 (http://support.illumina.com/sequencing/sequencing_software/casava.html) and RTA ver. 1.18.64].

#### Analysis of RNA sequencing data

For the read sequences resulting from the RNA sequencing, the adapter sequences (Truseq universal and indexed adapters) were removed using the Cutadapt software ver. 1.2.1 (https://cutadapt.readthedocs.io/en/v1.9.1/)^28^. The resulting reads were then aligned to the human reference genome (GRCh38) using TopHat aligner ver. 2.1.1 (https://ccb.jhu.edu/software/tophat/index.shtml) with the default options^29^. After the alignment, the mapped reads were counted for gene features (GTF file of GRCh38) using HTSeq ver. 0.6.1 (https://htseq.readthedocs.io/en/master/)^30^ and the estimated fragments per kilobase of transcript per million fragments were mapped (FPKM) using Cufflinks ver. 2.2.1 (http://cole-trapnell-lab.github.io/cufflinks/)^31^. The raw and normalized data were deposited with the Gene Expression Omnibus (GEO) database (GSE148402).

#### Identification of DEGs

To ensure statistical power in the identification of DEGs, we first selected ‘expressed’ genes as the ones with fragments per kilobase of exon model per million reads mapped (FPKM) values larger than 0 in at least half of the five PTB or FTB samples (n ≥ 3). To identify DEGs among the genes with meaningful expression in either PTB or FTB group, we further selected the genes with the maximum of the non-zero FPKM values larger than a cutoff of 1. The cutoff value was used to determine meaningfully expressed genes^32,33^. For these expressed genes, the read counts were normalized using the TMM normalization method^34^ in the edgeR package ver. 3.6 (https://bioconductor.org/packages/edgeR/)^35^. The log_2_ (read count + 1) were then normalized using the quantile normalization method^36^. For each gene, we calculated the T-statistic values and log_2_-fold-changes in the comparison of PTB versus FTB. We estimated empirical null distributions of the T-statistic values and log_2_-fold-changes by performing random permutations of the ten samples 300 times. Using the estimated empirical distributions, we computed the adjusted *p* values for the two tests for each gene and then combined these *p* values with Stouffer’s method^37^. Finally, we identified DEGs as the ones that had combined *p* values < 0.05, t-test *p* values < 0.05, and absolute log_2_-fold-changes > 0.58 (1.5-fold). For the analysis of the previous gene expression datasets, we collected the processed data from the GEO database (GSE46510 and GSE96083) and applied the same integrative statistical methods used for our data. The distribution of log_2_-fold-changes in GSE46510 and GSE96083 showed smaller variances than that in our data. For GSE46510 and GSE96083, we thus used the cutoffs as the mean of 10th and 90th percentiles and the mean of 2.5th and 97.5th percentiles of the empirical null distributions for log_2_-fold-changes, respectively (0.34 for GSE46510 and 0.50 for GSE96083).

#### GOBP enrichment analysis

To identify the cellular processes represented by the DEGs, we performed the enrichment analysis of GOBPs for the up- or downregulated genes using DAVID software ver. 6.8 (https://david.ncifcrf.gov/tools.jsp)^19^ and then selected the GOBPs with *p* value < 0.05 as the processes enriched by the up- or downregulated genes. We used the default *p* value from the EASE test in DAVID software, rather than a multiple testing-corrected *p* value (e.g., Benjamini–Hochberg *p* value), because we needed to ensure a sufficient list of GOBPs enriched by the small gene set (116 predictor genes) of the PREBIC PTB predictors for their comparisons with GOBPs enriched by the DEGs from our and previous mRNA expression datasets. The resulting GOBPs may include false positives due to the relaxed *p* value without the multiple testing correction. To remove potential false positives, we only selected GOBPs with the number of genes involved in the enriched GOBPs larger than 5 (i.e., count ≥ 5). We further focused on the overlapping “enriched GOBPs” between different gene sets (PREBIC PTB predictors, our DEGs, and DEGs identified from previous mRNA datasets). This analytical scheme helps us better interpret functional associations shared between molecular signatures identified from different PTB-related datasets, as decreasing the possibility to miss certain shared functional associations.

#### Reconstruction of network models

We first selected the up-regulated genes involved in T-cell activation-associated processes^38^ (lymphocyte activation and cytokine signaling) and the downregulated genes involved in the innate immunity-associated processes (innate immune response, toll-like receptor (TLR) signaling pathway, cytokine secretion, and granulocyte migration/chemotaxis). For the selected up- or downregulated genes, we then constructed a network model showing interactions among the selected genes using the protein–protein interactome databases^39–49^ and the interactions in Kyoto Encyclopedia of Genes and Genomes (KEGG) pathway database^50^. Key signaling molecules (gray nodes in the network models) were included in the network models to improve the connections among the selected genes, based on the KEGG pathways related to the network models (T-cell receptor [TCR], TNF, and NF-kappa B, TLR, PI3K-AKT, and MAPK signaling pathways, and Fc gamma R-mediated phagocytosis and regulation of actin cytoskeleton). The nodes in the network models were arranged according to the activation or repression information obtained from the KEGG pathway database and the previous literature.

#### qRT-PCR analysis

For qRT-PCR analysis, 1 μg of RNA was reverse transcribed to cDNA using SuperScript III reverse transcriptase (Invitrogen, Carlsbad, CA, USA) and RNasin (Promega, Madison, WI, USA) in a 20 μL reaction mixture. qRT-PCR was then performed in a 20 μL reaction mixture containing cDNA, 200 nM primers for each gene, SYBR Premix EX Taq (Takara Bio, Shiga, Japan), and ROX reference dye (Takara Bio) using a PRISM 7000 sequence detection system (Applied BioSystems, Foster City, CA, USA). Briefly, the samples were heated to 95 °C for 10 min and then amplified for 40 cycles at 95 °C for 15 s, and annealed at 62 °C for 1 min, followed by a dissociation stage at 95 °C for 15 s and 62 °C for 20 s per cycle. The quantity of each gene was calculated using the ΔΔCT method and based on the cycle threshold (CT) normalized against glyceraldehyde-3-phosphate dehydrogenase (GAPDH). The primer sequences used for qRT-PCR are listed in Table S4.

## Supplementary Information

Supplementary Information.

Supplementary Table S3.

## References

[1] Arzuaga BH, Lee BH (2011). Limits of human viability in the United States: a medicolegal review. Pediatrics.

[2] Organisation for Economic Co-operation and Development. OECD iLibrary. & WHO. 1 online resource 117 p (OECD/Korea Policy Centre, Seoul, 2016).

[3] Samuel TM (2019). Preterm birth: a narrative review of the current evidence on nutritional and bioactive solutions for risk reduction. Nutrients.

[4] Kwon EJ (2018). Risk factors for preterm birth in advanced maternal age. J. Korean Soc. Maternal Child Health.

[5] Liu L (2012). Global, regional, and national causes of child mortality: an updated systematic analysis for 2010 with time trends since 2000. Lancet.

[6] Bhutta AT, Cleves MA, Casey PH, Cradock MM, Anand KJ (2002). Cognitive and behavioral outcomes of school-aged children who were born preterm: a meta-analysis. JAMA.

[7] Luu TM, Rehman Mian MO, Nuyt AM (2017). Long-term impact of preterm birth: neurodevelopmental and physical health outcomes. Clin. Perinatol..

[8] Parets SE, Bedient CE, Menon R, Smith AK (2014). Preterm birth and its long-term effects: methylation to mechanisms. Biology (Basel).

[9] Hong JY (2020). Changes in the perinatal outcomes of twin pregnancies delivered at a tertiary referral center in Korea during a 24-year period from 1995 to 2018. Obstet. Gynecol. Sci..

[10] Menon R (2019). Initiation of human parturition: signaling from senescent fetal tissues via extracellular vesicle mediated paracrine mechanism. Obstet. Gynecol. Sci..

[11] Yoo JY (2018). Differential expression and methylation of integrin subunit alpha 11 and thrombospondin in the amnion of preterm birth. Obstet. Gynecol. Sci..

[12] Menon R (2011). Biomarkers of spontaneous preterm birth: an overview of the literature in the last four decades. Reprod. Sci..

[13] Heng YJ, Pennell CE, Chua HN, Perkins JE, Lye SJ (2014). Whole blood gene expression profile associated with spontaneous preterm birth in women with threatened preterm labor. PLoS ONE.

[14] Paquette AG (2018). Comparative analysis of gene expression in maternal peripheral blood and monocytes during spontaneous preterm labor. Am. J. Obstet. Gynecol..

[15] Chim SS (2012). Systematic identification of spontaneous preterm birth-associated RNA transcripts in maternal plasma. PLoS ONE.

[16] Bukowski R, Hankins GD, Saade GR, Anderson GD, Thornton S (2006). Labor-associated gene expression in the human uterine fundus, lower segment, and cervix. PLoS Med..

[17] Ngo TTM (2018). Noninvasive blood tests for fetal development predict gestational age and preterm delivery. Science.

[18] Chae S (2013). A systems approach for decoding mitochondrial retrograde signaling pathways. Sci. Signal..

[19] da Huang W, Sherman BT, Lempicki RA (2009). Systematic and integrative analysis of large gene lists using DAVID bioinformatics resources. Nat. Protoc..

[20] Knijnenburg TA (2019). Genomic and molecular characterization of preterm birth. Proc. Natl. Acad. Sci. USA.

[21] Arenas-Hernandez M (2019). Effector and activated T cells induce preterm labor and birth that is prevented by treatment with progesterone. J. Immunol..

[22] Luciano AA, Yu H, Jackson LW, Wolfe LA, Bernstein HB (2011). Preterm labor and chorioamnionitis are associated with neonatal T cell activation. PLoS ONE.

[23] Gomez-Lopez N, StLouis D, Lehr MA, Sanchez-Rodriguez EN, Arenas-Hernandez M (2014). Immune cells in term and preterm labor. Cell. Mol. Immunol..

[24] Allen LH (2000). Anemia and iron deficiency: effects on pregnancy outcome. Am. J. Clin. Nutr..

[25] Schuster J (2019). Effect of prematurity on genome wide methylation in the placenta. BMC Med. Genet..

[26] Stock O (2015). Chorioamnionitis occurring in women with preterm rupture of the fetal membranes is associated with a dynamic increase in mRNAs coding cytokines in the maternal circulation. Reprod. Sci..

[27] Gomez-Lopez N (2019). The cellular transcriptome in the maternal circulation during normal pregnancy: a longitudinal study. Front. Immunol..

[28] Martin M (2011). Cutadapt removes adapter sequences from high-throughput sequencing reads. EMBnet J..

[29] Trapnell C, Pachter L, Salzberg SL (2009). TopHat: discovering splice junctions with RNA-Seq. Bioinformatics.

[30] Anders S, Pyl PT, Huber W (2015). HTSeq—a Python framework to work with high-throughput sequencing data. Bioinformatics.

[31] Trapnell C (2010). Transcript assembly and quantification by RNA-Seq reveals unannotated transcripts and isoform switching during cell differentiation. Nat. Biotechnol..

[32] Mun DG (2019). Proteogenomic characterization of human early-onset gastric cancer. Cancer Cell.

[33] Wang K (2019). Multi-strategic RNA-seq analysis reveals a high-resolution transcriptional landscape in cotton. Nat. Commun..

[34] Robinson MD, Oshlack A (2010). A scaling normalization method for differential expression analysis of RNA-seq data. Genome Biol..

[35] Robinson MD, McCarthy DJ, Smyth GK (2010). edgeR: a Bioconductor package for differential expression analysis of digital gene expression data. Bioinformatics.

[36] Bolstad BM, Irizarry RA, Astrand M, Speed TP (2003). A comparison of normalization methods for high density oligonucleotide array data based on variance and bias. Bioinformatics.

[37] Hwang D (2005). A data integration methodology for systems biology. Proc. Natl. Acad. Sci. USA.

[38] Ashburner M (2000). Gene ontology: tool for the unification of biology. The Gene Ontology Consortium. Nat Genet.

[39] Bovolenta LA, Acencio ML, Lemke N (2012). HTRIdb: an open-access database for experimentally verified human transcriptional regulation interactions. BMC Genomics.

[40] Keshava Prasad TS (2009). Human Protein Reference Database—2009 update. Nucleic Acids Res..

[41] Licata L (2012). MINT, the molecular interaction database: 2012 update. Nucleic Acids Res..

[42] Orchard S (2014). The MIntAct project—IntAct as a common curation platform for 11 molecular interaction databases. Nucleic Acids Res..

[43] Patil A, Nakai K, Nakamura H (2011). HitPredict: a database of quality assessed protein-protein interactions in nine species. Nucleic Acids Res..

[44] Rolland T (2014). A proteome-scale map of the human interactome network. Cell.

[45] Rual JF (2005). Towards a proteome-scale map of the human protein-protein interaction network. Nature.

[46] Salwinski L (2004). The database of interacting proteins: 2004 update. Nucleic Acids Res..

[47] Stark C (2006). BioGRID: a general repository for interaction datasets. Nucleic Acids Res.

[48] Venkatesan K (2009). An empirical framework for binary interactome mapping. Nat. Methods.

[49] Yu H (2011). Next-generation sequencing to generate interactome datasets. Nat. Methods.

[50] Kanehisa M, Goto S (2000). KEGG: kyoto encyclopedia of genes and genomes. Nucleic Acids Res..

