# Supplementary material for: Integrative analysis of transcriptomic data for identification of T-cell activation-related mRNA signatures indicative of preterm birth
Source: Sci Rep. 2021 Jan 27;11:2392. doi: 10.1038/s41598-021-81834-z (PMC7841165; doi:10.1038/s41598-021-81834-z)
Supplement: Supplementary file 1 — Supplementary Information. [file 41598_2021_81834_MOESM1_ESM.docx]

**Integrative analysis of transcriptomic data for the identification of biomarkers associated with T-cell activation in preterm birth**

Jae Young Yoo^a,^**^†^**, Do Young Hyeon^b,^**^†^**, Yourae Shin^b,^**^†^**, Soo Min Kim^a^, Young-Ah You ^a,c,^**^*^**,

Daye Kim^d^, Daehee Hwang^b,*^ and Young Ju Kim^a,c,*^

^a^Department of Obstetrics and Gynecology, College of Medicine and Ewha Medical Institute, Ewha Womans University, Seoul 07804, Republic of Korea.

^b^School of Biological Sciences, Seoul National University, Seoul 08826, Republic of Korea.

^c^Ewha Medical Institute, Ewha Medical Center, Ewha Womans University, Seoul 07804, Korea.

^d^Internship in Ewha Womans University Mokdong Hospital, College of Medicine, Ewha Womans University, Seoul, 07804, Republic of Korea

**^†^**These authors contributed equally to this work.

This document includes Supplementary figures and tables.

**Supplementary Figures**

**
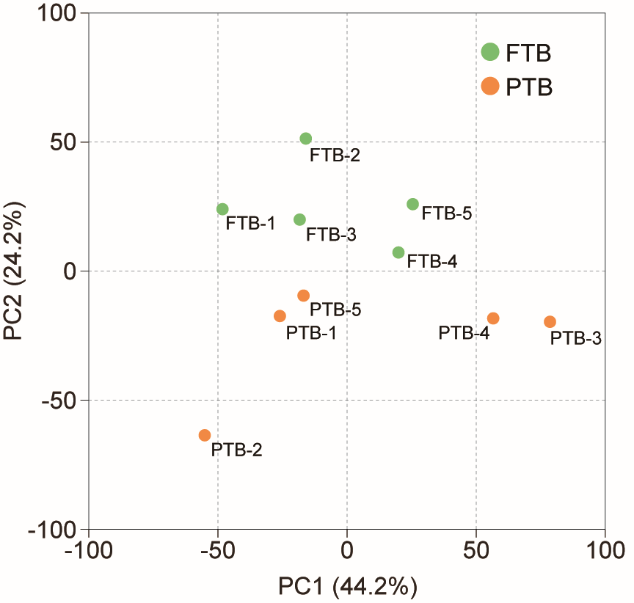
**

**Figure S1. Principal component analysis (PCA) of the samples in the discovery cohort using RNA-seq data.** The five PTB patients (PTB-1 to 5) and 5 FTB controls (FTB-1 to 5) were projected onto the first two principal components (PC1 and 2) that explained the 44.2 and 24.2% of the variance in the mRNA sequencing data, respectively. The projected values of the data for PTB-1 to 5 (orange) and FTB-1 to 5 (green) are displayed in a 2-d PCA score plot (PC1 versus PC2).

**Supplementary Tables**

**Table S1.** **Clinical characteristics of subjects in the discovery (A) and validation (B) cohorts.** Data were presented as Mean ± SE. * Student’s t-test, p < 0.05; ^†^χ^2^ test, p < 0.05. BMI, body mass index; PTL, Preterm labor; pPROM, Preterm premature rupture of membrane; and FT, Full term.

1. **Discovery cohort** (5 PTB patients and 5 FTB controls)

|  | Full term birth | Preterm birth | *p*-value |
| --- | --- | --- | --- |
|  | (*n* = 5) | ( *n* = 5) |  |
| Maternal age | 32.6 ± 1.9 | 34.0 ± 3.0 | 0.708 |
| Education, *n* (%) |  |  | 0.444 |
| Below high school | 0 ( 0.0) | 2 (40.0) |  |
| College or more | 5 (100) | 3 (60.0) |  |
| Parity |  |  | 0.469 |
| Nulliparous | 3 (60.0) | 2 (40.0) |  |
| Multiparous | 2 (40.0) | 3 (60.0) |  |
| Gravidity |  |  | 0.565 |
| 0 | 3 (60.0) | 4 (80.0) |  |
| ≥ 1 | 2 (40.0) | 1 (20.0) |  |
| Diagnosis |  |  |  |
| PTL |  | 2 (40.0) |  |
| PPROM |  | 3 (60.0) |  |
| FT | 5 (100) |  |  |
| WBC (ｘ10^3^/ml) | 10.09 ± 1.1 | 11.9 ± 1.0 | 0.249 |
| C-reactive protein | - | 2.0 ± 0.7 |  |
| Chorioamnionitis, *n* (%) |  |  | 0.048^†^ |
| Positive | - | 4 (80.0) |  |
| Negative | 5 (100) | 1 (20.0) |  |
| Antibiotics, *n* (%) |  |  | 0.048^†^ |
| Treatment | 1 (20.0) | 5 (100) |  |
| No | 4 (80.0) | - |  |
| Tocolytics |  |  | 0.048^†^ |
| Treatment | - | 4 (80.0) |  |
| No | 5 (100) | 1 (20.0) |  |
| Gestational age | 39.3 ± 0.3 | 31.6 ± 1.4 | 0.002^*^ |
| Birth weight (g) | 3297.5 ± 141.5 | 1764.0 ± 195.8 | <0.001^*^ |
| Gender, n (%) |  |  | 1.000 |
| Male | 5 (80.0) | 5 (80.0) |  |
| Female | 1 (20.0) | 1 (20.0) |  |
| APGAR 1min | 10.0 ± 0.0 | 6.8 ± 1.2 | 0.037^*^ |
| APGAR 5min | 10.0 ± 0.0 | 8.2 ± 7.3 | 0.040^*^ |

1. **Validation cohort** (83 PTB patients and 113 FTB controls)

| Items | Full term birth | Preterm birth | *p*-value |
| --- | --- | --- | --- |
|  | (n=113) | (n=83) |  |
| Maternal age | 33.4 ± 0.4 | 32.9 ± 0.5 | 0.410 |
| Education, *n* (%) |  |  | 0.022^†^ |
| Below high school | 10 ( 8.8) | 17 (20.5) |  |
| College or more | 103 (91.2) | 66 (79.5) |  |
| Pregnancy BMI | 26.4 ± 0.4 | 24.6 ± 0.4 | 0.001^*^ |
| Parity, *n* (%) |  |  | 0.886 |
| Nulliparous | 53 (46.9) | 38 (45.8) |  |
| Multiparous | 60 (53.1) | 45 (53.6) |  |
| Gravidity, *n* (%) |  |  | 0.143 |
| 0 | 61 (54.0) | 54 (65.1) |  |
| ≥ 1 | 52 (46.0) | 29 (34.9) |  |
| Diagnosis, *n* (%) |  |  | ^†^ |
| PTL |  | 55 (66.3) |  |
| PPROM |  | 28 (33.7) |  |
| FT | 113 (100) |  |  |
| WBC (ｘ10^3^/ml) | 10.28 ± 0.3 | 11.25 ± 0.5 | 0.078 |
| Gestational Age | 39.0 ± 0.1 | 31.6 ± 0.5 | 0.001^*^ |
| Mode of delivery, *n* (%) |  |  | 0.004^†^ |
| Vaginal | 73 (65.2) | 36 (43.9) |  |
| C-section | 39 (34.8) | 46 (56.1) |  |
| Chorioamnionitis, *n* (%) |  |  | 0.001^†^ |
| Positive | 5 ( 4.4) | 32 (38.6) |  |
| Negative | 108 (95.6) | 51 (61.4) |  |
| Antibiotics, *n* (%) |  |  | 0.006^†^ |
| Treatment | 48 (42.5) | 52 (62.7) |  |
| No | 65 (57.5) | 31 (37.3) |  |
| Tocolytics |  |  |  |
| Treatment | - | 37 (44.6) | 0.001^†^ |
| No | 113 (100) | 46 (55.4) |  |
| Birth weight (g) | 3146.5 ± 39.7 | 1869.2 ± 102.0 | 0.001^*^ |
| Gender, *n* (%) |  |  | 0.464 |
| Male | 57 (52.8) | 48 (58.5) |  |
| Female | 51 (47.2) | 34 (41.5) |  |
| APGAR 1min | 9.6 ± 0.1 | 6.7 ± 0.4 | 0.001^*^ |
| APGAR 5min | 9.9 ± 0.0 | 8.3 ± 0.3 | 0.001^*^ |

**Table S2. Summary statistics of mRNA sequencing data.** Mapping and unique mapping rates are presented with the numbers of mapped and uniquely mapped reads.

| Sample | Number of measured reads | | Number of mapped reads | | | Number of uniquely mapped reads | | |
| --- | --- | --- | --- | --- | --- | --- | --- | --- |
|  | Pre-filtered reads | Filtered reads | mapped | unmapped | mapping rate | uniquely mapped | multiply mapped | unique mapping rate |
| FTB control-1 | 51,106,840 | 50,982,858 | 44,551,468 | 6,431,390 | 0.87 | 42,257,294 | 2,294,174 | 0.95 |
| FTB control-2 | 59,754,562 | 59,640,083 | 53,038,615 | 6,601,468 | 0.89 | 34,789,650 | 18,248,965 | 0.66 |
| FTB control-3 | 54,852,888 | 54,734,464 | 46,974,706 | 7,759,758 | 0.86 | 44,021,632 | 2,953,074 | 0.94 |
| FTB control-4 | 56,062,150 | 55,947,561 | 49,090,055 | 6,857,506 | 0.88 | 45,222,621 | 3,867,434 | 0.92 |
| FTB control-5 | 47,075,662 | 46,980,472 | 41,068,052 | 5,912,420 | 0.87 | 38,022,939 | 3,045,113 | 0.93 |
| PTB patient-1 | 53,212,272 | 53,090,732 | 46,667,111 | 6,423,621 | 0.88 | 44,211,248 | 2,455,863 | 0.95 |
| PTB patient-2 | 55,078,820 | 54,958,490 | 48,204,200 | 6,754,290 | 0.88 | 44,792,173 | 3,412,027 | 0.93 |
| PTB patient-3 | 54,530,194 | 54,409,346 | 46,865,473 | 7,543,873 | 0.86 | 42,343,462 | 4,522,011 | 0.90 |
| PTB patient-4 | 53,201,274 | 53,083,471 | 46,545,258 | 6,538,213 | 0.88 | 43,369,010 | 3,176,248 | 0.93 |
| PTB patient-5 | 60,142,810 | 60,013,424 | 52,298,229 | 7,715,195 | 0.87 | 49,045,998 | 3,252,231 | 0.94 |

**Table S3. DEGs between PTB and FTB.** For each of up- or down-regulated genes in PTB compared to in FTB, gene symbol and description, Ensemble Gene ID, and Entrez ID are shown together with combined P-value, adjusted P-value from t-test, and log_2_-fold-change of its expression level in PTB with respect to that in FTB.

See TableS3.xlsx excel file.

**Table S4.** **List of primers used for real-time PCR.** CYLD: CYLD lysine 63 deubiquitinase TFRC: transferrin receptor, SRC: SRC proto-oncogene, non-receptor tyrosine kinase, RIPK2: receptor interacting serine/threonine kinase 2, SMAD3: SMAD family member 3 and GAPDH: glyceraldehyde-3-phosphate dehydrogenase.

| Gene name | Primer category | Primer sequence (5' → 3') |
| --- | --- | --- |
| CYLD | Forward | GCA ACC TCA TGC AGT TCT |
|  | Reverse | AAA CCT TGA CCA CGA CCT |
| TFRC | Forward | GAC GCG CTA GTG TTC TTC T |
|  | Reverse | AAC CGG GTA TAT GAC AAT GG |
| SRC | Forward | ACT ATG AGT CTA GGA CGG AG |
|  | Reverse | CTG TGT TGT TGA CAA TCT GG |
| RIPK2 | Forward | TTT GGG AAT TTG CAA TGA GC |
|  | Reverse | AAG GAG GAG TCA TAT TGT GC |
| SMAD3 | Forward | GGG GTT GGA CTT TCC TTC |
|  | Reverse | CAG CAG AAG TTT GGG TTT C |
| GAPDH | Forward | TGG GCT ACA CTG AGC ACC AG |
|  | Reverse | AAG TGG TCG TTG AGG GCA AT |
